# Study of the role of epidermal growth factor on lung fluid transport in rabbits with acute lung injury caused by endotoxin

**DOI:** 10.3892/etm.2012.670

**Published:** 2012-08-16

**Authors:** BINOU YANG, WEIQING HUANG, JIEYUN HAN, ZIJING LIANG

**Affiliations:** 1 National Hepatobiliary and Enteric Surgery Research Center of Ministry of Health, Central South University, Changsha, Hunan 410008;; 2 The First Affiliated Hospital of Guangzhou Medical College, Guangzhou, Guangdong 510120, P.R. China

**Keywords:** epidermal growth factor, endotoxin, acute lung injury, lung fluid transport

## Abstract

The aim of this study was to investigate the effect of epidermal growth factor (EGF) on the lung fluid transport of rabbits with acute lung injury caused by endotoxin and evaluate its therapeutic action. A total of 24 rabbits were randomly divided into control, simple acute lung injury (ALI) and EGF only treatment groups. ALI rabbit models were constructed by the administration of endotoxin (lipopolysaccharide, LPS) and subsequent treatment with EGF. Arterial partial pressure of oxygen, lung pathomorphological changes and wet/dry weight (W/D) of the left lobe of lung tissue were observed at various time points. Results showed that following treatment with EGF, the breathing status of the rabbits continued to improve. An increase was noted in PaO_2_ at 12 h after EGF treatment and 24 h later PaO_2_ had significantly increased. A marked decrease was observed in the value of W/D and the exudation was reduced. The extrinsic EGF decreased the exudation of pulmonary capillaries and improved lung water transport. Our findings verified that epidermal growth factor had repaired the effect of ALI through continuous 48-h observation. Therefore, the present study demonstrated the therapeutic action of EGF.

## Introduction

Acute lung injury (ALI) is one of the clinically familiar critical emergencies. Studies have shown that ALI has a high mortality rate and that 40–50% of patients succumb within several days after onset of the disease (1) The pathogenesis of ALI is complicated and remains to be elucidated. ALI is characterized by rapid alveolar injury, inflammation, cytokine induction and neutrophil accumulation (2). Although early events in the pathogenesis of ALI have been defined, the mechanisms underlying its resolution are unknown. At present, various studies have started to explore the molecular mechanisms involved in the pathogenesis and progression of ALI (2–3).

Studies on the pathological characteristics of ALI have shown that it is an inflammatory reaction that induces lesions in pulmonary capillary endothelial cells and the alveolar epithelium (4). Pulmonary capillary hyperpermeability leads to pneumonedema and the formation of a transparent film accompanied by pulmonary interstitial fibrosis (5). The inflammation of lung tissue mediated by neutrophilic granulocytes is the pathological foundation of pulmonary capillary hyperpermeability and pneumonedema. Epidermal growth factor (EGF) is an effective enhancer that stimulates various types of tissue cell to multiple degradation. Previous studies on the biological effect of EGF in the lung demonstrated that EGF promotes the DNA synthesis of numerous types of cells in the lung, including fibroblast, epithelial and granular alveolar cells (6). EGF also promotes water transportation in the lung, which aids in the treatment of lung injury. EGF augmented ALI by improving the transmembrane transport of alveolar type II cells, resulting in the acceleration of alveolar liquid clearance. Additionally, exogenous EGF adjusts and controls pulmonary surfactant generation.

Currently there are no effective methods to cure ALI. To investigate the effect of extrinsic EGF on lung fluid transport in rabbits with ALI caused by endotoxin and evaluate its therapeutic action, we constructed ALI rabbit models using endotoxin and dripped EGF into trachea to investigate the curative effect of EGF on ALI.

## Materials and methods

### Grouping of the laboratory animals

A total of 24 healthy rabbits (provided by Guangzhou Medical College Animal Experimental Center, Guanzhou, China) of either gender were selected. The weight of the animals varied from 1.90 to 2.50 kg. The rabbits were randomly divided into three groups; control, simple ALI and EGF treatment (endotoxin-induced ALI + EGF treatment) groups. Each group was divided into four subgroups according to the time points of 1, 12, 24 and 48 h.

### Reagents

Endotoxin (*E. coli* O111:B4) was produced by Sigma (St. Louis, MO, USA) at a density of 0.1 mg/ml. The EGFs were produced by Sigma. The reagents heparin, napental (3%) and physiological saline used for the EGF solution were obtained from Shanghai No. 1 Biochemical and Pharmaceutical Co., Ltd.

### Construction of animal models with endotoxin

The acute lung injury animal model was constructed by injecting endotoxin lipopolysaccharide (LPS) intravenously into the rabbit through the ear vein. Pathological indices confirmed the accuracy of the animal model. Thus, following treatment with LPS, the volume of the lung enlarged and dark red patch foci of unequal sizes on the surface and light red or white frothy liquid overflow from the section were observed. Leukocyte infiltration was observed in lung tissue through light microscopy. Pulmonary interstitial and alveolar edema, as well as pulmonary hemorrhage were evident.

### Intervention of EGF

EGF was administered to the rabbits shortly after the endotoxin had caused ALI. The method used was as follows: rabbits were immobilized, EGF (100 μg/kg) was taken up with a 1-ml injector and thinned to 1 ml with physiological saline, a syringe needle was introduced into the windpipe through the glottis, the liquid was sprayed quickly into the windpipe through the syringe needle and the animals were returned to feeding.

### Determination of the arterial partial pressure of oxygen (PaO_2_)

The change in rabbit respiration, heart rate, color of lips and other symptoms was observed. At each time point, 1–2 ml of blood was phlebotomized from the rabbit ear central artery and a blood gas analysis was performed immediately. Heparin was used to moisten the wall of the injector.

### Determination of the water content of lung tissues

The rabbits were sacrificed following anaesthesia with 3% napental. The left lung was dissociated and the wet weight (W) was measured. At 24 h after anhydration under 0.06 ×70°C conditions, the dry weight (D) was measured. The water content of the lung tissues was then recorded as W/D.

### Pathomorphological observation of lung tissue

The left inferior lung lobe was removed, fixed and embedded. It was then sliced, dyed with hematoxylin and eosin stain (H&E) and observed under a light microscope.

### Statistical analysis

The experimental data were processed by SPSS 13.0 and presented as the mean ± standard deviation. Analysis of variance was used to the compare groups and the Student’s t-test was used to compare the rabbit PaO_2_ and ratio of W/D in the three groups at four periods of time. P<0.05 was considered to indicate a statistically significant result.

## Results

### Characteristics of the rabbits

The rabbits in the control group were in a healthy state, the breathing rhythm was even and the color of the skin and mucous membranes were normal. In the simple ALI group, the breathing rate increased and the skin and mucous membranes developed cyanosis 10 min after the injection of endotoxin. The breathing and heart rate became reduced and cyanosis decreased in the EGF treatment group 30 min after therapy.

### Arterial partial pressure of oxygen (PaO_2_)

Compared with the control group, PaO_2_ in the rabbits which had been injected with endotoxin 1 h before decreased from 16.24±0.87 kPa to 6.99±1.45 kPa. PaO_2_/FiO_2_ decreased from 580 to 249, while the X-ray examination showed that there was diffuse leakage in both lungs. The abovementioned symptoms were standard of ALI. Following treatment with EGF, the breathing state continued to improve. PaO_2_ increased to 13.78±0.62 kPa 24 h later (Table I).

### W/D of lung tissues

The W/D of the simple ALI group was much higher than that of the control group, indicating that there was a large quantity of liquid overflow in the alveoli following endotoxin-induced lung injury. At 24 h after the treatment with EGF the pulmonary edema was reduced to a certain degree from 8.68±0.43 to 5.20±0.74 (Table II).

### Lung tissue under light microscopy

**Figure 1:**
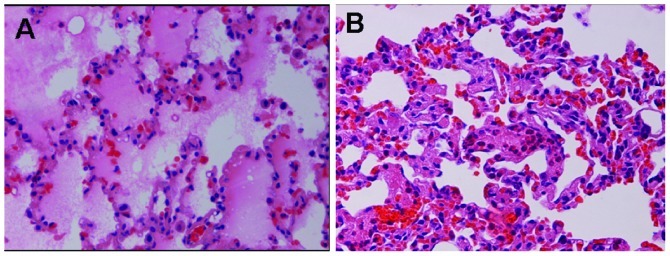
(A) Lung tissue of simple ALI group in 24 h. (B) Lung tissue of EFG treatment group. Samples were stained with hematoxylin and eosin; magnification, ×200.

The rabbit lungs in the normal control group appeared to be a uniform pink, with a smooth envelope and good flexibility, with no liquid overflow from the section. Results obtained from light microscopy showed that the lung tissue was structurally complete, the alveolar cavity was clear and the alveolar septum had no special manifestation such as edema or inflammation. The lungs in the simple ALI group had a dirty red appearance, particularly the two lower sides of the lungs. There was a large quantity of bloody frothy fluid overflowing from the section. Destruction of the alveolar wall, edema and hemorrhage in interstitial alveoli, and a decrease of alveoli and diffuse neutrophilic granulocyte infiltration were observed under the light microscope (Fig. 1). The lesions in the EGF treatment group had a lighter appearance to a certain degree. Results of the light microscopy showed that the majority of the alveolar wall was restored, edema in interstitial alveoli was alleviated and only a small quantity of neutrophilic granulocyte infiltration was present (Fig. 2).

## Discussion

Studies have shown that ALI has a high mortality rate and 40–50% of patients succumb to multiple organ failure, not hypoxia (1). The pathogenesis of ALI is complicated and has yet to be clearly explained. The majority of patients often succumb within several days following onset of the disease. ALI remains a difficult clinical problem. At present, studies have started to explore the molecular mechanisms involved in the pathogenesis and progression of ALI (2–3).

Since ALI and gram-negative bacteria cause sepsis, the endotoxin-induced injury ALI model is used in ALI pathogenesis research. Endotoxins, including LPS, are constituents of gram-negative bacterial cell walls. Through intravenous injection or tracheal inhalation endotoxins can be used to construct ALI animal models (4,7–9). The construction of this model is key to the endotoxin or sepsis as it induces alveolar capillary damage and pulmonary edema. Fiber proteins, platelets and white blood cells are able to cause a microthrombosis and increase in pulmonary arterial pressure. The process of platelet aggregation releases vascular-active substances, complement activation and aggregation of polymorphonuclear neutrophil, and in theory, is similar to the characteristics of the clinical cases (6).

PME cells, inflammatory factors and damaged epithelial intercellular signaling are three main areas that play a role in the pathogenesis of ALI (10). Studies on the pathological characteristics of ALI have shown that it is the inflammatory response that is responsible for the development of the lesion of pulmonary capillary endothelial cells and alveolar epithelium, while pulmonary capillary hyperpermeability leads to pneumonedema and transparent film formation accompanied by pulmonary interstitial fibrosis. The inflammation of lung tissue mediated by neutrophilic granulocytes is the pathological basis of pulmonary capillary hyperpermeability and pneumonedema.

EGF is a powerful cell division promoting factor and stimulates the body responsible for the organization of cell division and proliferation (11). At present, the use of EGF to treat ALI has been studied in animal models. Studies have confirmed that exogenous EGF treatment of ALI of rat alveolar type II cells is able to significantly increase cell proliferation. Thus, the exogenous epidermal growth factor is capable of protecting alveolar epithelial function, so as to reduce the lung injury (12). Studies on the biological effect of EGF in the lung have demonstrated that EGF promotes DNA synthesis of numerous types of cells in the lung, such as fibroblast, epithelial and granular alveolar cells. EGF also promotes water transportation in the lung, which aids in the treatment of lung injury (13). EGF increases alveolar liquid clearance rate (ALC) by improving the ion transmembrane transport of AT-II, resulting in the acceleration of liquid clearance in alveoli. Exogenetic EGF adjusts and modulates pulmonary surfactant generation (14,15). Results of the present study have shown that group W/D was significantly higher than the normal control group following endotoxin-induced lung injury. Additionally, there was extensive fluid overflow from the alveoli. The W/D value decreased significantly 24 h after EGF treatment, due to pulmonary edema, with a certain degree of ease (16–18).

The present has shown how EGF stimulates vascular endothelial cell migration and proliferation from a molecular pathology point of view (19). The results demonstrated that EGF promotes the restoration of the lung vessels to repair lung, reduces pulmonary edema and improves the reversal of ALI. Following treatment with EGF, the breathing and heart rate had improved (20). An increase in PaO_2_ was observed after 12 h, a marked increase in PaO_2_ was noted 24 h later and further improvement was evident 48 h later. Pathological examination and the W/D measurement revealed that the lung injury in the EGF treatment group was alleviated to a certain extent. The EGF treatment group W/D was 5.86±0.78 and significantly different compared with the simple ALI group W/D which was 8.23±0.94. Continuous observation for 48 h confirmed the curative effect of EGF on ALI. EGF may therefore provide a new treatment of ALI.

The literature reports that EGF promotes the proliferation of type II alveolar cells and water transportation in the lung, although its mechanism is complicated. The present study only shows the therapeutic action of EGF via observation of the pathology, improvement in symptoms and blood gas analysis. However, the use of EGF requires more study before it can be safely used in the clinic.

## Figures and Tables

**Table I:** Rabbit PaO_2_ (kPa) in three groups at four periods of time.

Groups	T1	T12	T14	T48
Normal control	16.24±0.87	16.94±0.82	16.43±0.78	16.65±0.80
Simple ALI	6.99±1.45^a^	6.84±1.42^a^	6.67±1.48^a^	6.75±1.32^a^
EGF treatment	10.78±0.46^b^	11.16±0.54^b^	13.78±0.62^b^	13.84±0.56^b^

a Compared with the normal control group, P<0.05;

b compared with the simple ALI group, P<0.05.

**Table II:** Ratio of W/D in three groups of rabbits at four periods of time.

Groups	T1	T12	T24	T48
Normal control	4.41±0.46	4.39±0.50	4.31±0.52	4.28±0.55
Simple ALI	8.23±0.64^a^	8.53±0.58^a^	8.68±0.43^a^	8.85±0.44^a^
EGF treatment	5.86±0.78^b^	5.23±0.65^b^	5.20±0.74^b^	5.06±0.72^b^

a Compared with the normal control group, P<0.05;

b compared with the simple ALI group, P<0.05.
